# Ten-month-old infants’ neural tracking of naturalistic speech is not facilitated by the speaker’s eye gaze

**DOI:** 10.1016/j.dcn.2023.101297

**Published:** 2023-09-29

**Authors:** Melis Çetinçelik, Caroline F. Rowland, Tineke M. Snijders

**Affiliations:** a Department of Experimental Psychology, Utrecht University, Utrecht, the Netherlands; b Max Planck Institute for Psycholinguistics, Nijmegen, the Netherlands; c Donders Institute for Brain, Cognition and Behaviour, Radboud University, Nijmegen, the Netherlands; d Cognitive Neuropsychology Department, Tilburg University, Tilburg, the Netherlands

**Keywords:** Neural tracking of speech, Eye gaze, Social cues, Speech processing, Speech entrainment, EEG power

## Abstract

Eye gaze is a powerful ostensive cue in infant-caregiver interactions, with demonstrable effects on language acquisition. While the link between gaze following and later vocabulary is well-established, the effects of eye gaze on other aspects of language, such as speech processing, are less clear. In this EEG study, we examined the effects of the speaker’s eye gaze on ten-month-old infants’ neural tracking of naturalistic audiovisual speech, a marker for successful speech processing. Infants watched videos of a speaker telling stories, addressing the infant with direct or averted eye gaze. We assessed infants’ speech-brain coherence at stress (1–1.75 Hz) and syllable (2.5–3.5 Hz) rates, tested for differences in attention by comparing looking times and EEG theta power in the two conditions, and investigated whether neural tracking predicts later vocabulary. Our results showed that infants’ brains tracked the speech rhythm both at the stress and syllable rates, and that infants’ neural tracking at the syllable rate predicted later vocabulary. However, speech-brain coherence did not significantly differ between direct and averted gaze conditions and infants did not show greater attention to direct gaze. Overall, our results suggest significant neural tracking at ten months, related to vocabulary development, but not modulated by speaker’s gaze.

## Highlights

• Ten-month-old infants’ neural tracking of speech was assessed with a naturalistic audiovisual paradigm.
• Infants showed reliable neural tracking of the stress and syllable patterns in audiovisual speech.
• Infants’ neural tracking of speech was not modulated by a speaker’s use of eye contact, nor was their attention to the speaker.
• Increased neural tracking of the syllable rate was related to better later language outcomes.

## 1 Introduction

Infants’ early experiences with language usually occur in social contexts, during face-to-face interactions with their caregivers. In these interactions, infants are exposed to a range of social cues in addition to the linguistic input, and gradually learn to use these cues to understand the communicative intent of their communicative partner (Csibra and Gergely, 2009). In fact, many prominent theories of language development highlight the role of such social factors (e.g. Hollich et al., 2000; Kuhl, 2007; Tomasello, 2000, Tomasello, 2003), and argue that children’s ability to understand their communicative partner’s intentions, and their responsiveness to joint attention, play a role in language development (Brooks and Meltzoff, 2005, Brooks and Meltzoff, 2008, Carpenter et al., 1998, Hirotani et al., 2009, Kuhl, 2007, Morales et al., 2000a, Morales et al., 2000b).

Among these social cues in communication, eye gaze stands out as an important, attentionally-salient ostensive cue. Infants show a sensitivity to eye gaze, both in the form of mutual gaze and gaze following, from early on. Newborns (Farroni et al., 2002), as well as older infants (Farroni et al., 2002, Farroni et al., 2007), prefer to look at faces with direct gaze as opposed to averted gaze. Given infants’ early sensitivity for, and selective attention to, gaze cues, Natural Pedagogy Theory suggests that the use of ostensive cues in communication, such as mutual gaze, signals the communicative intent of the social partner to the infant, which may optimise information transfer between the infant and the adult (Csibra and Gergely, 2009, Senju and Csibra, 2008). This facilitation may be realised through direct eye gaze evoking high-excitability oscillatory periods for optimal information encoding (Wass et al., 2020).

A similar theory in the language development literature is Kuhl’s “social gating” hypothesis (Conboy et al., 2015, Kuhl, 2007), which suggests that social interaction is crucial for infants’ speech processing and phonemic discrimination. On this view, language learning is strongly facilitated in social settings when information is provided by live tutors, but not from passive listening to language input. However, despite a large literature on the role of social factors in infants’ early vocabulary development, studies on the effect on early speech processing are scarce (Çetinçelik et al., 2021). In particular, only a few studies systematically looked at which social cues, such as eye gaze, influence infants’ speech processing, and their findings are by no means conclusive. For instance, Lloyd-Fox et al. (2015) found that 6-month-old infants’ neural activation in response to speech was enhanced, especially in brain regions that are involved in processing social communication when the speaker provided direct gaze while speaking (see Holler et al., 2014 for similar results in adults). Similarly, Leong, Byrne et al. (2017) reported that brain-to-brain synchrony between infant-adult dyads was larger when the adult addressed the infant with direct gaze. However, this effect was not demonstrated in another study looking at naturalistic infant-caregiver interactions (Marriott Haresign et al., 2023). Thus, while it seems plausible that direct gaze may influence infants’ speech processing, we need more studies that systematically investigate the effect of eye gaze to draw firm conclusions.

One promising way to assess successful speech processing in infants is by studying the neural tracking of speech. Neural tracking refers to the process by which neural oscillations track the dynamic patterns of the speech signal at multiple levels of linguistic information (Giraud and Poeppel, 2012). Recent studies have demonstrated that infants, like adults, can track the amplitude envelope of naturalistic speech at multiple rates (Attaheri et al., 2022a, Attaheri et al., 2022b, Jessen et al., 2019, Kalashnikova et al., 2018, Menn et al., 2022, Menn et al., 2022, Ortiz Barajas et al., 2021, Tan et al., 2022). In infant-directed speech, the stress and syllable frequencies are particularly emphasised, as the amplitude modulation spectra of infant-directed speech contain peaks around the prosodic stress (around 2 Hz), syllable (around 5 Hz) and phoneme (around 20 Hz) frequencies (Leong and Goswami, 2015). This enhanced expression of prosodic stress might have functional implications for language development. The stressed syllables, marked by auditory “edges” (Doelling et al., 2014) may serve as cues to word onset in infant-directed speech, especially in languages that have word-initial lexical stress, such as English and Dutch (Cutler and Carter, 1987, Vroomen et al., 1998). These salient cues in continuous speech can aid listeners in word segmentation (Johnson and Jusczyk, 2001, Jusczyk et al., 1999), which has been linked to individual differences in vocabulary development (Junge et al., 2012, Kidd et al., 2018, Kooijman et al., 2013).

Indeed, it has been argued that neural tracking of speech, especially at the stress and syllable rates, might be an important underlying mechanism for early speech processing and vocabulary development (Goswami, 2019). Relatedly, recent studies demonstrated a link between infants’ neural tracking abilities and later vocabulary skills (Attaheri et al., 2022a, Attaheri et al., 2022b, Menn et al., 2022, Ní Choisdealbha et al., 2022). That said, most studies on infants’ neural tracking either used auditory-only paradigms, or looked at neural tracking of speech without taking the multimodal and social nature of naturalistic communication into account (but see Menn, Michel et al., 2022 for a naturalistic paradigm; and Attaheri et al., 2022a, Attaheri et al., 2022b; Menn, Ward et al., 2022; Ní Choisdealbha et al., 2022; Tan et al., 2022 for audiovisual speech). As discussed earlier, one important aspect of naturalistic communication is the use of eye gaze by social partners, which might act as a cue for infants to allocate their attention to what is worthwhile for them to attend in their environment, such as a speaker. In adult studies, neural tracking of speech has been linked to attentional mechanisms. Adult listeners tracked the speech of a speaker better when they successfully selectively attended to one speaker over two simultaneously presented speech streams, referred to as the “cocktail party effect” (Ding and Simon, 2012, O’Sullivan et al., 2015, Power et al., 2012). Thus, in adult listeners, neural tracking of speech may, at least partially, rely on attention. Similarly, social cues, such as the speaker’s eye gaze, might help infants direct attention to relevant speech stimuli, which might facilitate neural tracking of speech as a result of enhanced attention.

There is some evidence that infants’ neural speech tracking abilities is enhanced by the presence of ostensive social cues, but only from a very few studies. Infant-directed speech (IDS) is one such cue that caregivers frequently use to address infants, which signals that information is intended and relevant for the infant receiver (Csibra, 2010). Kalashnikova et al. (2018) demonstrated that infant-directed speech (IDS) led to greater tracking of speech in seven-month-old infants compared to adult-directed speech (ADS) (though note that this enhancement could be due to bottom-up mechanisms arising from the low-level features of IDS, such as greater pitch range and more regularised rhythm, as well as/instead of top-down processes such as greater attention to IDS (Cooper and Aslin, 1990)). In a design using naturalistic face-to-face interaction between parents and nine-month-old infants, Menn et al. (2022) also found that IDS facilitated infants’ neural speech tracking of the prosodic stress rate but also found, in a subsequent control analysis, that this facilitation was not modulated further by parents’ use of mutual gaze (note though that, given their focus on the comparison between IDS and ADS, parents’ use of eye contact was not systematically manipulated). Although not specifically testing the effects of eye gaze but those of visual speech cues, Tan et al. (2022) demonstrated that overall attention to the speaker’s face did not relate to individual differences in neural tracking of audiovisual speech, neither in five-month-old infants, nor in four-year-old children.

There are even fewer studies directly manipulating eye gaze. In a study looking at infant-adult dyads’ levels of brain-to-brain synchrony as a function of eye gaze while the adult was singing nursery rhymes, Leong, Byrne et al. (2017) also reported a control analysis of speech-brain synchrony, where no differences were identified between the direct and averted gaze conditions. However, Çetinçelik et al. (2023) did report that infants processed single words differently as a function of the speaker’s eye gaze. In a familiarisation and test paradigm, infants were familiarised with passages that contained target words, either with direct or with averted gaze, and then were presented with the target word and a novel word in isolation. Although infants showed the ERP word recognition effect for the target word both after the direct and averted gaze familiarisation, direct gaze led to a differential processing of familiarised single words, especially over midline and right frontal areas. However, it is still not clear whether eye gaze facilitates infants’ processing of multimodal, continuous speech.

Thus, the first aim of the current study was to test whether ten-month-old infants show greater neural tracking of speech, indexed by speech-brain coherence, when the speaker used mutual (direct) gaze to address the infant when speaking, compared to averted gaze, as a function of enhanced attention to speech with eye contact. We used speech-brain coherence, reflecting the consistency of the phase difference between the brain activity and the speech amplitude envelope at a given frequency, to assess neural tracking, because it directly measures the synchronicity between the oscillations and the speech envelope (Peelle et al., 2013). Our second aim was to explore whether infants show greater frontal EEG power in the theta band (3–6 Hz) for direct gaze compared to averted gaze. It has been suggested that ostensive cues such as IDS facilitate infants’ attention (Cooper and Aslin, 1990), which will then be reflected in changes in EEG power at the theta band (3–6 Hz in infants), as increases in frontal and midline theta power have been linked to endogenous sustained attention (Orekhova et al., 2006), anticipation of receiving information from a social partner (Begus et al., 2016), and the social nature of the interaction/stimuli (Jones et al., 2015). Hence, we predicted that a speaker’s use of eye gaze would lead to greater EEG power in frontal and midline regions, as a result of enhanced attention to social speech. Our third aim was to assess the functional relevance of neural tracking by investigating whether neural tracking is related to later language. It has been argued that successful tracking of the low-frequency information (stress and syllable rhythm) in speech may give infants an advantage in speech encoding and word segmentation from continuous speech, which then lead to better later vocabulary outcomes (Attaheri et al., 2022a, Attaheri et al., 2022b, Menn et al., 2022, Ní Choisdealbha et al., 2022).

To this end, we focused on ten-month-old infants, as these infants are expected to still rely on prosodic cues in speech (e.g. for word segmentation; Kooijman et al., 2009; Männel and Friederici, 2013). Moreover, ten-month-olds are expected to have gone through the “nine-month revolution” (Tomasello, 1995, Tomasello, 1999), a developmental stage in which infants are thought to become increasingly responsive and motivated to share attention with others, and to learn to recognise the communicative intentions of social partners.

## 2 Methods

This study was preregistered (https://aspredicted.org/blind.php?x=VSG_ZNB). The preregistration also included an assessment of infants’ word segmentation abilities, but this is reported in a different paper (Çetinçelik et al., 2023). Any deviations from the pre-registered analysis pertaining to the results reported in the current paper are listed in Supplementary Materials A.

### 2.1 Participants

Participants were 50 Dutch 10-month-old infants (mean age = 308.3 days, age range = 291–326 days; 28 female). An additional 40 infants were tested but were excluded because they did not meet the inclusion criteria due to having more than four noisy or flat channels (*n =* 5), lack of sufficient artefact-free trials (see details below; *n =* 26), technical issues (*n* = 4) or refusal to wear the cap and fussiness (*n* = 5). All infants were born full-term, were normally developing, and were raised in monolingual Dutch-speaking households. Caregiver(s) reported no neurological or language problems in the immediate family. Participants were recruited from the Nijmegen Baby and Child Research Center database. The study was approved by the Ethical Board of Social Sciences, Radboud University, Nijmegen. Caregiver(s) gave written informed consent for the study, in accordance with the Declaration of Helsinki. Families were offered a choice between 20 Euros and a book for their participation.

### 2.2 Materials

Materials consisted of blocks of audio-visual familiarisation sentences (four sentences per block) followed by isolated audio-only test words (see Table 1 for an example block, and Supplementary Materials D, Supplementary Table 1 for the full set of materials).

**Table 1:** An example of an experimental block (English translations in parentheses, with the familiarised target word in bold).

**Familiarisation phase**
1. Er zitten **cello’s** in het orkest. *(There are cellos in the orchestra.)* 2. Goede **cello’s** zijn van hout gemaakt. *(Good cellos are made of wood.)* 3. Ik hoorde vanochtend **cello’s**. *(I heard cellos this morning.)* 4. Met de pauken spelen vaak de **cello’s** mee. *(The cellos often play along with the timpani.)*
**Test phase**
1. **Cello’s** *(cellos)* 2. Tuba’s *(tubas)*

#### 2.2.1 Familiarisation stimuli

For the familiarisation phase, 30 combinations of sentences (“familiarisation blocks”) were created, each comprising four sentences in which a target word was repeated. Target words were low-frequency disyllabic trochaic Dutch words. Three versions of these familiarisation blocks were formed using the same sentences, but with different target words in each version. This resulted in 90 familiarisation blocks, and 360 sentences in total. The sentences consisted of 8–12 syllables.

The familiarisation stimuli were 90 videos of a female Dutch actor, who was speaking either with direct gaze or averted gaze. During stimulus recording, the actor sat face on to the camera, looked at a picture of an infant and was instructed to use infant-directed speech. In order to ensure acoustic consistency of the speech properties across conditions, videos were recorded simultaneously from three angles: (1) speaker looking directly at the camera in the middle (direct gaze); (2) speaker’s head averted at an approximately 20-degree angle to the left (averted gaze); and (3) speaker’s head averted at an approximately 20-degree angle to the right (averted gaze). Stimuli were recorded using Adobe Audition. Videos were edited using Adobe Premier Pro, and the audio of the familiarisation stimuli were processed and normalised to 70 dB using Praat (Boersma and Weenink, 2021). The familiarisation stimuli had a mean sentence duration of 3197 ms (*SD* = 507 ms) and an inter-sentence interval of approximately 1500 ms (*M* = 1501.8 ms, *SD* = 71.2 ms). The mean duration of the familiarisation blocks was 18.9 s

To identify the frequency ranges of the linguistic units in the stimuli, familiarisation stimuli were annotated and analysed using Praat (Boersma and Weenink, 2021). The duration of all stressed syllables, syllables, words and sentences were transcribed, and mean frequencies and frequency ranges were calculated for each unit, excluding inter-sentence intervals, but including pauses between utterances. In our stimuli, stressed syllables occurred at a rate of 0.87–1.72 Hz, syllables at 2.62–3.57 Hz and words at 1.52–2.58 Hz. Based on these frequencies, we selected the following rates for the speech-brain coherence analyses: 1–1.75 Hz for the stressed syllable rate and 2.5–3.5 Hz for the syllable rate.

#### 2.2.2 Test stimuli

The 90 experimental words were recorded separately in isolation, and were further normalised to 70 dB using Praat (Boersma and Weenink, 2021). The mean word duration was 911 ms (*SD* = 127 ms). Only audio stimuli were used in the test phase of the experiment, which did not form part of the analysis reported in the current study.

#### 2.2.3 Language outcome tests

Infants’ vocabulary knowledge was assessed using the Dutch version of the MacArthur-Bates Communicative Development Inventories (CDI), a caregiver-report measure of receptive and expressive vocabulary (Zink and Lejaegere, 2002; adapted from Fenson et al., 1993). Caregivers reported the items their child “understands” and “understands and says”, which index infants’ receptive and expressive vocabulary sizes respectively. At 10 months, caregivers filled in the N-CDI-1 (maximum possible score = 103 for each category). Parents were invited to fill in the N-CDI-2 at 18 months to test infants’ subsequent vocabulary development (maximum possible score = 112 for each category).

### 2.3 Design

Participants saw a maximum of 60 experimental blocks, each consisting of a familiarisation phase and a test phase (The analyses reported in the current paper only used the audiovisual familiarisation phase). In the familiarisation phase, infants watched a video of the actor reciting four sentences with one repeated target word. The familiarisation phase was followed by the test phase, consisting of two audio-only single words, one of which was the repeated target (familiar) word, and the other one was a novel control word (order counterbalanced). The test words were presented auditorily, without any attention getters. An example experimental block is illustrated in Table 1.

The second half of the experiment (blocks 31–60) consisted of the same familiarisation videos as in the first half but using a different control word in the test phase. Blocks were presented in a pseudo-randomised order, with a minimum of 10 intervening blocks between the first presentation of the same familiarisation video and the second. Three different versions (A, B, C) of the experiment were created out of the 90 blocks, resulting in 30 blocks in each version. The target word in one version was used as the control word in the other two versions. Each participant was presented with two out of three versions (i.e. if an infant was familiarised with version A, they would hear the control words from version B in the first half, and control words from version C in the second).

Each participant was presented with a maximum of 30 blocks in the direct gaze condition, and 30 blocks in the averted gaze condition, with the gaze condition changing every 2–3 blocks. To account for the potential confound of the visual features of one side of the face affecting the experimental outcomes, both the speaker gazing at left and right were used as the averted gaze condition, counterbalanced between participants, but kept constant within participants. The familiarisation versions (A/B/C) and the sequence of the blocks were pseudo-randomised and counterbalanced.

### 2.4 Procedure

The study required two experimenters. The first experimenter briefed the caregivers about the study while the infants played on a play mat, and the second experimenter pre-gelled the EEG cap to minimise setup time, and then fitted the cap to the infants’ head. Electrode impedances were checked, and more gel was added if necessary. After capping, the infants sat in their caregivers’ lap in an electrically shielded and sound-attenuated testing booth, approximately 70 cm away from a 24-inch display monitor. The videos were displayed at the centre of the screen (20 ×20 cm). Audio stimuli were presented over two loudspeakers at approximately 65 dB.

Stimuli were presented using Presentation (Version 20.2, Neurobehavioral Systems, Inc., Berkeley, CA, www.neurobs.com). The experiment started with an attention getter, followed by two silent 10-second baseline videos of the speaker with direct and averted gaze with a 1000 ms inter-stimulus interval (ISI), to accustom the infants to the actor. Then, the first experimental block was presented. Each experimental block consisted of the presentation of the familiarisation video first, and then, after approximately 1500 ms, the presentation of the target and control words with an approximately 1500 ms ISI (order counterbalanced). The inter-trial interval between two experimental blocks, that is, the offset of the test phase of one block and the onset of the familiarisation video of the next block was 3000 ms. Every four to five blocks, short attention getters were presented in a pseudo-randomised order.

During the session, caregivers listened to masking music through noise-cancelling closed-ear headphones. Caregivers were instructed not to interact with the infants, but only offer silent toys or breadsticks if they became restless. The experimenters were seated outside of the booth to run the experiment and EEG acquisition. If the infants became fussy, a short break was taken, and infants were presented with a silent cartoon video. The experiment was stopped if the infants became distressed or disengaged from the screen for an extended period. The sessions were video recorded for offline coding of infant behaviour and attention to the screen. The whole session including preparation lasted about an hour, and the experiment lasted about 25–30 min. In line with the institution’s COVID-19 measures, the experimenters and caregivers wore face masks during the session, and the experimenters additionally wore face shields while fitting the cap.

### 2.5 Looking behaviour

Infants’ looking behaviour during the presentation of each familiarisation block were manually coded using ELAN Version 6.3 (2022). Infants’ looks to the screen and looks away from the screen during the familiarisation videos were coded frame-by-frame.

Following our EEG analysis pipeline, the looking time data were segmented into four second epochs with a one-second sliding window to match them with the EEG trials. Then, infants’ attention for each four-second epoch were computed by calculating the proportion of their looking time to the screen per epoch. Epochs were excluded if the infant attended to the screen for less than 25 % (1 s) of the 4-second epoch. This cut-off is similar to the thresholds reported in other EEG and eye-tracking studies (Tan et al., 2022). Note that we also conducted an exploratory analysis in which we included trials during which infants looked at the screen for 50 % or more of the trial duration (see Supplementary Materials C), which yielded the same pattern of results as reported below.

### 2.6 EEG recordings and data processing

EEG was recorded from 32 electrodes (actiCAP with active Ag/AgCl electrodes) positioned according to the extended 10–20 system, using BrainAmp DC and Brain Vision Recorder software (Brain Products GmbH, Germany). FCz was used as the online reference. EEG was recorded from Fp1, Fp2, F7, F3, Fz, F4, F8, FC5, FC1, FC2, FC6, T7, C3, Cz, C4, T8, TP9, CP5, CP1, CP2, CP6, TP10, P7, P3, Pz, P4 and P8. Two additional electrodes were placed directly on the mastoid bones (“TP9L”, “TP10L”) as potential reference electrodes in addition to the mastoid electrodes in the cap (TP9, TP10). EOG was recorded from the electrode above (Fp1) and an additional electrode placed below the left eye, and from the two electrodes at the outer canthi of the eyes (FT9, FT10). Data were recorded with a sampling rate of 500 Hz, using an online time cut-off of 10 s and a high cut-off of 1000 Hz. Impedances were typically kept under 25 Ω.

EEG data were processed and analysed in MATLAB (version 2020b) using the Fieldtrip toolbox for EEG/MEG-analysis (Oostenveld et al., 2011). First, the complete dataset was pre-processed to provide as much data as possible for Independent Component Analysis (ICA; Makeig et al., 1996). Data were filtered with a Hamming windowed Butterworth high-pass filter of 0.1 Hz (−12 dB/oct) and a low-pass filter of 30 Hz, and segmented into 1-second snippets for artefact rejection. Data were visually inspected and bad channels and data segments with flat channels or high amplitude artefacts (exceeding 150 μV for EEG channels, 250 μV for EOG channels) were excluded. Next, eye movement and single channel noise components were identified using Independent Component Analysis (ICA; Makeig et al., 1996), infomax ICA (Bell and Sejnowski, 1995). Eye and single channel noise components were identified by visual inspection of the components and data, and were marked to remove these components from the data in the next analysis step (below).

#### 2.6.1 Speech-brain coherence pre-processing

For the speech-brain coherence analyses, raw EEG data were re-segmented into 4-second epochs using a 1-second sliding window, starting from the onset up until the offset of the familiarisation videos. Four-second data epochs were used to get a frequency resolution of .25 Hz, while overlapping epochs were used to avoid unnecessary data loss (similar to Menn et al., 2022; Menn et al., 2022; Ríos-López et al., 2020). These raw data trials were again filtered from 0.1 to 30 Hz, and eye movement components and single-channel noise components previously identified with ICA based on visual detection of component morphology (see above) were removed from the epochs. On average, 2.8 eye (range: 1–5) and 4.02 single-channel noise components (range: 1–8) were removed. In addition, two posterior channels (P7 and P8) were removed because these channels were identified as too noisy across many datasets. The epoched data were then re-referenced to the linked mastoids (TP9L and TP10L, or TP9 and TP10, or a bilateral combination), and demeaned. If referencing to linked mastoids was not possible due to the linked reference electrode being noisy, a single mastoid reference was used (for six infants).

The acoustic envelope of the familiarisation stimuli was computed using a Hilbert transform with a second-order Butterworth filter, downsampled to 500 Hz to match the sampling rate of the EEG data and cut into the same 4-second epochs as the EEG data. The EEG data were combined with the respective acoustic envelope, and trials with amplitudes exceeding ± 150 μV were rejected. Moreover, trials during which infants attended to the screen for less than 25 % of the trial duration (less than one second in a four-second epoch), previously determined by the looking time analysis, were excluded. The mean number of trials with at least 25 % looking (regardless of inclusion in the final dataset based on artefact rejection) was 510.6 (SD = 146.7). To get a reliable coherence estimate (Bastos and Schoffelen, 2016), only infants who had a minimum of 30 artefact-free epochs in each condition, thus 60 artefact-free epochs overall, were included in the final dataset. This resulted in an overall average of 215.8 included artefact-free epochs with at least 25 % looking (overall range = 69–664; Direct: M= 108.6, range = 31–332; Averted: M = 107.1, range: 32–332). Noisy channels in the remaining datasets (mean number of repaired channels = 0.9; range = 0–3) were repaired using spherical spine interpolation (Perrin et al., 1989).

Finally, EEG data and the speech envelope were Fourier-transformed from 1 to 10 Hz with a frequency resolution of .25 Hz, capturing the most important linguistic units in our stimuli. Then, coherence between the speech envelope and the EEG signal was calculated for each channel-speech signal combination, using the following formula, where S_xy_ (ω) is the cross-spectral density between the speech envelope (x) and the EEG signal (y) at frequency ω, and S_xx_ (ω) and S_yy_ (ω) are the power spectra of the speech envelope and the EEG signal (Bastos and Schoffelen, 2016; Rosenberg et al., 1989):

$${\mathrm{coh}}_{\mathrm{xy}}\left(\omega \right)=\frac{|S_{\mathrm{xy}}\left(\omega \right)|}{\sqrt{S_{\mathrm{xx}}\left(\omega \right)S_{\mathrm{yy}}\left(\omega \right)}}$$

This resulted in one coherence value between 0 and 1, reflecting the consistency of the phase difference between the two signals, amplitude envelope and brain activity, at a given frequency (Peelle et al., 2013).

### 2.7 Data analysis

#### 2.7.1 Speech-brain coherence analysis

First, to establish the presence of neural tracking, observed speech-brain coherence was compared to surrogate data. Surrogate data was created by shuffling the speech envelope across epochs and computing the average coherence of the shuffled envelope and the EEG data over 100 permutations, regardless of the experimental condition. Then, using non-parametric cluster-based randomisation tests (Maris and Oostenveld, 2007) with 1000 permutations (10,000 permutations if the initial p-value was close to the significance threshold), speech-brain coherence to observed data was compared to surrogate data, assessing all electrodes with a single test. This was first conducted over the whole 1–10 Hz frequency range, and then separately averaged over the stimulus-driven frequency ranges of interest, which are the stressed syllable (1–1.75 Hz) and syllable frequencies (2.5–3.5 Hz). The stressed syllable and syllable rates were investigated as these acoustic cues are argued to be the most pronounced linguistic units in the amplitude envelope of infant-directed speech and most relevant for early language development (Leong et al., 2017). Then, to test the effect of the speaker’s gaze on infants’ speech-brain coherence, infants’ speech-brain coherence in the two gaze conditions were compared to each other in the stressed syllable and syllable frequencies, over all electrodes.

For subsequent analyses, speech-brain coherence (overall and per condition) was z-score transformed using the mean and the standard deviation of the surrogate data in the respective condition to estimate coherence bias (Bastos and Schoffelen, 2016; see Vanden Bosch der Nederlanden et al., 2022 for a similar approach). Then, the standardised coherence values across all included electrodes in the frequency bands of interest (stressed syllable and syllable) were averaged, resulting in one coherence value per frequency rate for each infant.

#### 2.7.2 Looking times

To examine differences in attention to screen between the two conditions, infants’ looking times were compared with paired-samples t-tests, separately for the 4-second epochs that are included in the subsequent EEG analyses, and also for all trials, regardless of inclusion.

#### 2.7.3 Power analysis

We explored infants’ mean theta power (3–6 Hz) to assess whether direct gaze during speech is more attentionally salient for infants. For the theta frequency band, we selected the 3–6 Hz range, as this range has been most consistently reported in previous infant studies (e.g. Jones et al., 2015, Jones et al., 2020; Saby and Marshall, 2012; van der Velde et al., 2021), with a peak around 4.4 Hz (Meyer et al., 2022, Orekhova et al., 2006). To this end, we calculated the absolute EEG power for the same 4-second epochs included in the speech-brain coherence analyses using fast Fourier transformations in 0.25 Hz frequency steps from 1 to 10 Hz. Then, we compared the EEG power in the direct gaze condition to the averted gaze condition in the 3–6 Hz frequency band, using cluster-based permutation, assessing all electrodes without averaging over frequencies. Note that the theta band frequency narrowly overlaps with the syllable frequency band (2.5–3.5 Hz), which might have attenuated differences in theta band power between the two conditions due to common speech input, but this effect should be minimal given the slight overlap (3–3.5 Hz).

#### 2.7.4 Relationship between neural tracking and vocabulary development

Infants’ receptive and expressive vocabulary scores at 10 and 18 months were obtained from the parent-reported measures on the N-CDI. Data from seven infants were missing at 18 months, resulting in 43 infants who had data at both 10 and 18 months. Raw vocabulary scores were converted into proportion scores (individual score divided by the total number of possible vocabulary items per measure). To investigate the relationship between neural tracking and subsequent vocabulary development, linear regression models were fit separately to the receptive and expressive vocabulary scores at 18 months, using the *stats* package in R (version 4.2.2; R R Core Team, 2022). In both models, infants’ receptive or expressive vocabulary at 18 months was the dependent variable. Infants’ z-score transformed speech-brain coherence values at the stressed syllable and syllable rates, and their receptive or expressive vocabulary scores at 10 months, respectively, were entered as predictors. The 10-month receptive or expressive vocabulary was included as a predictor to control for infants’ vocabulary development at the time of the recording. This allows us to test whether neural tracking is related to growth in vocabulary between 10 and 18 months.

## 3 Results

### 3.1 Speech-brain coherence

To test for the presence of speech-brain coherence, observed speech-brain coherence was first compared to speech-brain coherence in surrogate data with a shuffled speech envelope, in the whole 1-to-10 Hz frequency range, without averaging over electrodes or frequencies. One large positive cluster emerged which encompassed all electrodes and frequencies from 1 to 10 Hz (cluster *p*_*corrected*_ *=*.002), which incorporates the stressed syllable, syllable and word rates.

**Fig. 1:**
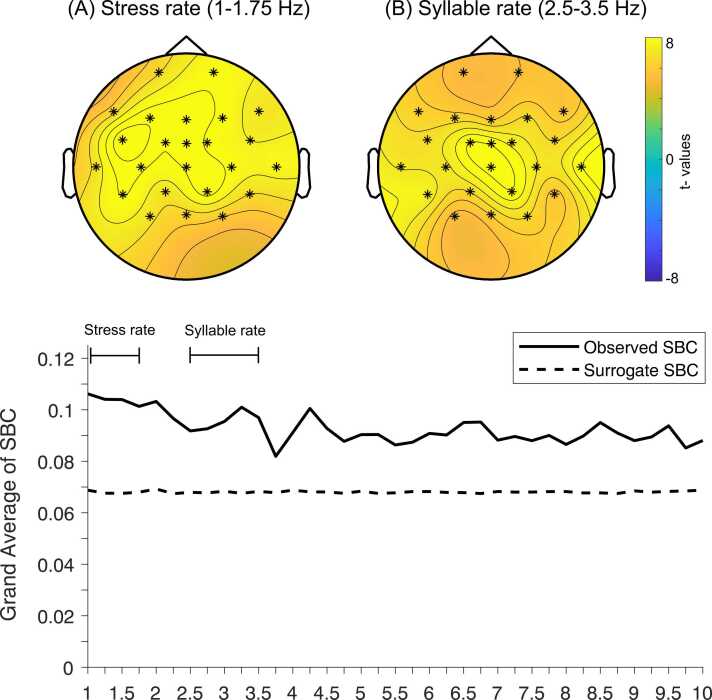
Overall Speech-Brain Coherence. Top row: Scalp topography of the coherence difference between overall observed SBC and SBC to surrogate data at (A) stress and (B) syllable frequency rates, showing the t-values of the comparison. Electrodes that form the significant cluster are highlighted with stars. Bottom row: overall SBC and SBC to surrogate data, averaged over all electrodes.

Then, observed speech-brain coherence and surrogate data were compared at the frequency ranges of interest, namely the stress (1–1.75 Hz) and syllable (2.5–3.5 Hz) frequencies. This comparison yielded significant positive clusters at both the stressed syllable and syllable rates (cluster *p*_*corrected*_ *=*.002 for both rates), including all electrodes (Fig. 1).

### 3.2 Speech-brain coherence and gaze effects

**Fig. 2:**
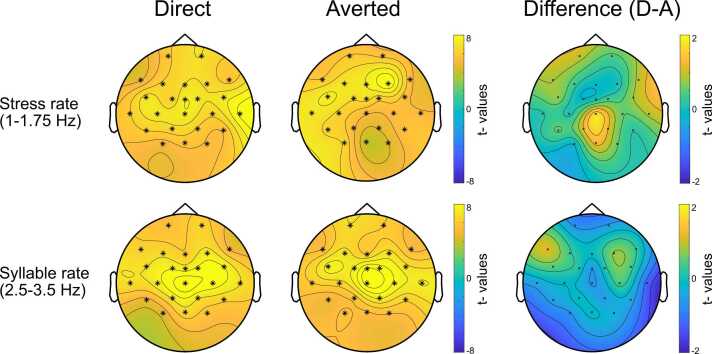
Scalp topography of speech-brain coherence in the Direct gaze (left column), Averted gaze conditions (middle column) and the difference between the Direct and Averted gaze conditions (right column). The top row illustrates coherence at the stress rate (1–1.75 Hz), and the bottom row illustrates coherence at the syllable rate (2.5–3.5 Hz). The topographies in the left and middle columns show the t-values of the comparison between real versus surrogate data in the two conditions, and the right column shows the comparison of the Direct and Averted conditions. Note the different scales used for the colour bar in the left and middle columns (t-values between −8 and 8) and the difference figure on the right (t-values between −2 and 2). Cluster electrodes involved in the significant clusters for real versus surrogate data (left and middle columns) are marked with stars.

Speech-brain coherence was higher in observed than in surrogate data in the stress and syllable rates in both experimental conditions, Direct and Averted gaze. For both conditions, one large positive cluster was identified in both frequency ranges (all cluster *p*_*corrected*_ *=*.002), over all electrodes (Fig. 2, left and middle columns).

To assess the effects of the speaker’s gaze on infants’ speech-brain coherence, we ran cluster-based permutation tests to compare speech-brain coherence in the two experimental conditions, Direct gaze and Averted gaze. First, the frequency range from 1 to 10 Hz was assessed for differences in the two conditions. This test identified three positive and five negative clusters, none of which was significant after correcting for multiple comparisons (*p* of the largest positive cluster =.71, *p* of the largest negative cluster =.92. A positive cluster indicates higher SBC with Direct gaze compared to Averted gaze, and negative cluster vice versa). Then, tests were performed separately in the pre-defined stress and syllable rates by averaging over these frequency rates, which did not yield any clusters, indicating that no differences were identified between conditions at these frequency ranges. The differences in coherence between the Direct and Averted gaze conditions are shown in Fig. 2 (right column).

### 3.3 Looking times

**Fig. 3:**
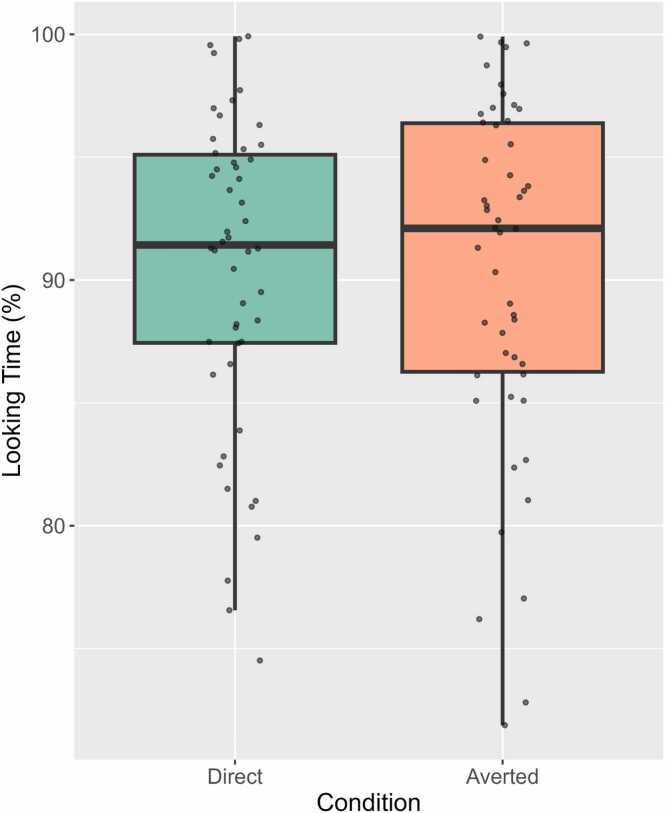
Proportion of infants’ mean looking times (percentages, calculated by the average proportion of looking to the screen during the 4-second epochs) in the included trials, for the Direct and Averted gaze conditions. The length of the boxes represents the interquartile range, the whiskers denote lower and upper values within 1.5 interquartile range, and the individual data points show each infant’s mean looking times averaged over trials.

A paired-samples t-test showed that infants’ mean looking times in the Direct and Averted gaze conditions in the included 4-second trials were not significantly different (*t*(49) = −0.08, *p* = .94; Direct: *M*= 3.62 s, *SD* = .26 s; Averted: 3.62 s, *SD* = .28 s). Fig. 3 illustrates the mean proportion of attention to videos as percentages for both conditions (see Supplementary Materials B, Supplementary Figure 1 for the comparison of all trials, including the trials that were excluded from analyses).

### 3.4 Power

The comparison of absolute EEG power in the Direct and Averted gaze conditions in the theta (3–6 Hz) frequency range resulted in two positive clusters (meaning that power to Direct gaze is larger than Averted gaze) and one negative cluster (meaning that power to Averted gaze is larger than Direct gaze), but these clusters did not survive multiple comparison correction (*p* of the largest positive cluster *=*.062, at 5–5.5 Hz; *p* of the largest negative cluster *=*.25; 10,000 permutations). Thus, infants’ absolute EEG power in the theta band was not significantly different between the Direct gaze and Averted gaze conditions.

### 3.5 Neural tracking and vocabulary development

The correlations between the predictor variables in both models are shown in Table 2. Although some predictors showed moderate correlations, multicollinearity was not a problem (VIFs < 1.3).

**Table 2:** Means, standard deviations, and correlations with confidence intervals of the predictor variables in both models. The mean and standard deviation values for the vocabulary measures are reported as proportions.

Variable	*M*	*SD*	1	2	3
1. SBC stress rate	1.71	0.97			
2. SBC syllable rate	1.47	0.86	.25		
			[−.05,.51]		
3. Receptive voc. 10 months	0.26	0.18	-.02	.40**	
			[−.31,.29]	[.11,.63]	
4. Expressive voc. 10 months	0.02	0.03	.25	.03	.39**
			[−.05,.51]	[−.28,.32]	[.10,.62]

*Note*. Values in square brackets indicate the 95 % confidence interval for each correlation. ***p < .001, **p < .01, *p* < *.05.

1: Speech-brain coherence at the stress rate (z-score)

2: Speech-brain coherence at the syllable rate (z-score)

3: Receptive vocabulary at 10 months

A regression model was fitted to infants’ receptive vocabulary scores at 18 months, with the z-score transformed speech-brain coherence values at the stressed syllable and syllable rates and their 10-month receptive vocabulary scores as predictors. There was significant model fit (*F*(3, 39) = 8.40, *p* < .001; Table 3) but the effect was mainly driven by 10-month receptive vocabulary predicting 18-month receptive vocabulary (*β* = 0.62, *SE* = 0.15, *t* = 4.29, *p* < .001), due to a strong relationship between infants’ receptive vocabulary scores at the two time points. None of the other main effects were significant.

**Table 3:** The results of the linear regression models with the z-score transformed speech-brain coherence (SBC) at the stress and syllable rates and 10-month receptive vocabulary (proportion) as predictors, and receptive vocabulary at 18 months (proportion) as the outcome measure.

	**Receptive vocabulary 18 months**		
*Predictors*	*β [95 % CI]*	*SE*	*p*
(Intercept)	0.43 [0.31 – 0.56]	0.06	<.001***
SBC stress rate	-0.02 [−0.07 – 0.03]	0.03	.396
SBC syllable rate	0.01 [−0.05 – 0.08]	0.03	.655
Receptive voc. 10 months	0.62 [0.33– 0.92]	0.15	<.001***
R^2^ / R^2^ adjusted	0.392 / 0.346		

Note: ***p < .001, **p < .01, *p* < *.05.

We also fitted a regression model to the expressive vocabulary data at 18 months, with the z-score transformed speech-brain coherence at both rates and infants’ 10-month expressive vocabulary scores as predictors. As testing the model assumptions of model with the 18-month expressive vocabulary score as the outcome variable suggested heteroscedasticity (non-constant variance *p* = .007), the outcome variable was square-root transformed. This model also showed a significant model fit (*F*(3, 39) = 5.44, *p* = .003; Table 4), but this time speech-brain coherence at the syllable rate significantly predicted expressive vocabulary at 18 months (*β* = 0.09, *SE* = 0.04, *t* = 2.51, *p* = .016). This suggests that infants with higher speech-brain coherence at the syllable rate at 10 months had larger expressive vocabularies when assessed eight months later, even after taking 10-month expressive vocabulary into account. However, tracking at the stress rate did not significantly predict later expressive vocabulary development. Furthermore, there was a significant main effect of expressive vocabulary at 10 months (*β* = 2.75, *SE* = 0.95, *t* = 2.90, *p* = .006).

**Table 4:** The results of the linear regression models with the z-score transformed speech-brain coherence (SBC) at the stress and syllable rates, and 10-month expressive vocabulary (proportion) as predictors, and expressive vocabulary at 18 months (proportion; square-root transformed) as the outcome measure.

	Expressive **vocabulary 18 months**		
*Predictors*	*β [95 % CI]*	*SE*	*p*
(Intercept)	0.20 [0.06 – 0.35]	0.07	.007**
SBC stress rate	0.00 [−0.06 – 0.07]	0.03	.966
SBC syllable rate	0.09 [0.02 – 0.16]	0.04	.016*
Expressive voc. 10 months	2.75 [0.83 – 4.67]	0.95	.006**
R^2^ / R^2^ adjusted	0.295 / 0.241		

Note: ***p < .001, **p < .01, *p* < *.05.

## 4 Discussion

In this study, we examined ten-month-old Dutch-learning infants’ neural tracking of audio-visual infant-directed speech, and tested whether neural tracking is facilitated by a speaker’s use of direct eye gaze as an ostensive cue in communication. We predicted that infants would track the speech rhythm at the stress and syllable rates, which, in our stimuli, had a frequency of 1–1.75 Hz and 2.5–3.5 Hz respectively. Furthermore, we hypothesised that infants would have increased speech-brain coherence, indexing enhanced neural tracking, and larger frontal EEG power in the theta band, indicating higher attention, when speech is accompanied by direct gaze as opposed to averted gaze. Finally, we predicted that infants’ neural tracking abilities would be positively related to their later vocabulary development.

Regarding neural tracking of speech, we found that ten-month-old infants showed neural tracking of the speech amplitude envelope at multiple rates, including at the predicted stress and syllable frequencies, at all tested electrodes. First, we observed a large cluster that emerged between the whole frequency range that we assessed, from 1 to 10 Hz. This range encompassed the stress, syllable, and word frequencies in our stimuli. Assessing stress and syllable rates specifically, we found reliable speech-brain coherence of the speech rhythm, suggesting that, at ten months, the infant brain is already able to track relevant regularities in the amplitude envelope of the speech signal. Overall, our speech-brain coherence findings are in line with the emerging literature on infants’ neural tracking of naturalistic speech and songs (Attaheri et al., 2022a, Attaheri et al., 2022b, Jessen et al., 2019, Kalashnikova et al., 2018, Menn et al., 2022, Menn et al., 2022, Ortiz Barajas et al., 2021, Tan et al., 2022).

Contrary to our main prediction, we did not observe a significant difference between infants’ speech-brain coherence in the direct gaze and averted gaze conditions. This means that neural tracking was not facilitated by the speaker’s use of eye gaze to convey ostensive communication, consistent with the results by Leong, Byrne et al. (2017). One implication of this unanticipated finding is that social cues in communication, such as mutual gaze, in fact, might not facilitate speech processing at all. However, this explanation seems unlikely since this is contrary both to previous studies and a number of theories that suggest a key role of sensitivity to social cues both more broadly in learning and language development (Csibra and Gergely, 2009; Hollich et al., 2000; Kuhl, 2007; Tomasello, 2003), and in speech processing in particular, where enhanced activation in response to infant-directed speech was observed when accompanied by direct gaze (Lloyd-Fox et al., 2015). Moreover, one study looking at infants’ word segmentation with a familiarisation-then-test paradigm, using the same experimental conditions as in this study, found differential processing of familiar words, as indicated by differences in the ERP word familiarity effect (Çetinçelik et al., 2023). Thus, we suggest a more narrow-scope explanation; that while social cues do facilitate some aspects of speech processing, infants’ neural tracking of speech specifically is not modulated by the speaker’s use of some social cues, such as eye gaze.

Indeed, previous research has shown that neural tracking of speech is present from birth, and does not depend on social, and possibly other attentional, cues. It has been demonstrated that even newborns track the phase and amplitude of both native and non-native languages (Ortiz Barajas et al., 2021). Furthermore, speech tracking in newborns is often measured while they are sleeping, showing that tracking can also occur in the absence of attention (Ortiz Barajas et al., 2021). During naturalistic interactions, Menn et al. (2022) also found that infants’ neural tracking was unaffected by whether or not the caregiver established eye contact with the infant. Similarly, in a study with five-month-old infants, Tan et al. (2022) reported that infants’ attention to the speaker’s face was not related to their neural tracking of speech, although a positive correlation was observed for adults’ attention levels and their neural tracking of visual-only speech. Thus we suggest that in the first year of life, neural tracking may be a predominantly stimulus-driven mechanism, facilitated by bottom-up factors such as the temporal regularities in the speech rhythm (Kalashnikova et al., 2018). However, in some circumstances, such as social interaction (Leong et al., 2017), or more challenging listening conditions such as the presence of multiple speakers (Zion Golumbic et al., 2013) top-down cues might enhance neural tracking. This point will be examined in more detail below when we compare our results to studies that employed live interaction paradigms.

Bottom-up cues play an important role in infant speech processing in general (Hollich et al., 2005; Lewkowicz, 2010; Yeung and Werker, 2013) and neural tracking of speech in particular (Tan et al., 2022). Important cues are visual speech cues, which are cues that are conveyed by the speaker’s lip and mouth movements. Crucially, the movement of the speaker’s mouth provides an important cue for neural tracking, as the opening and closing of the lips is tightly linked to the speech rhythm, especially at the syllable rate (Zoefel, 2021), and provides reliable predictive information about the rhythmic patterns of speech. Moreover, it has been suggested that infants attend selectively to the speaker’s mouth rather than the eyes between 6 and 12 months (Lewkowicz and Hansen-Tift, 2012). Given that the visual speech cues were equally visible in this study, regardless of the speaker’s gaze direction, these visual speech cues might have bottom-up facilitated neural tracking of speech further. This might explain the lack of a gaze effect. In other words, in the presence of bottom-up cues such as visual speech cues, top-down cues such as infants’ attention to the speaker’s gaze direction, may simply be unnecessary for successful neural tracking.

That said, real-life speech input is much noisier than in our study, where clearly articulated infant-directed speech was produced by a single speaker. Rather, speech is typically accompanied by environmental background noise, and multi-speaker interference, which also holds for speech provided to infants (McMillan and Saffran, 2016). Research with adults has shown that under more challenging listening conditions, such as a multi-talker environment, top-down factors modulate neural tracking of speech, such that adults’ neural tracking of an attended speaker was higher than an unattended speaker (Zion Golumbic et al., 2013). Thus, social cues might still play a role in facilitating infants’ speech perception in complex conditions such as speech in noise, which develops during childhood (Bertels et al., 2023), or the presence of multiple competing speakers, possibly by directing infants’ attention to the speaker and thereby enhanced neural tracking of the attended speaker’s speech input. Further research is necessary to explore this idea.

As well as finding no effect of eye gaze on neural tracking, we also did not identify significant differences in infants’ attention to the videos when the speaker addressed them with direct or averted gaze. Behaviourally, infants’ looking times to both conditions were similar, as was their absolute EEG power in the theta range, which has been linked to differences in attention, especially in endogenous and anticipatory sustained attention (Begus et al., 2015, Begus et al., 2016, Jones et al., 2015, Orekhova et al., 1999). Overall, no significant in theta band power were found, neither over the frontal and midline regions as predicted, nor over other electrode sites. This suggests that infants were paying attention to the speaker, regardless of the speaker’s gaze direction. These results are similar to that of Kalashnikova et al. (2018) who found no significant differences in 7-month-olds theta power when listening to IDS and ADS, although other studies have demonstrated that IDS lead to enhanced frontal theta compared to other control conditions such as absence of speech (Orekhova et al., 2006), or comparing vowels pronounced using IDS versus ADS (Zhang et al., 2011).

While this finding was unexpected, as previous studies reported that infants looked preferentially and longer to pictures of faces with direct eye contact (Farroni et al., 2002, Farroni et al., 2007), this discrepancy might be explained by the age of the infants tested (10 months). There is a developmental shift in the first year of life, in which early infants’ preference for direct gaze, when their primary mode of communication is dyadic (i.e. face-to-face with the caregiver), slowly transitions into the perception of eye gaze as a dynamic and interactive social signal (Carpenter et al., 1998). With this transition, infants may come to understand that the partner’s social attention does not necessarily require moment-to-moment eye contact, and that their gaze may also be other-directed (Senju et al., 2008). In fact, in naturalistic caregiver-child interactions, eye gaze directed at the communicative partner during the whole course of interaction is rather rare, and both infants and caregivers frequently shift their attention between objects in the environment (e.g. toys) and the other partner (Abney et al., 2020, Wass et al., 2018, Wass et al., 2018, Yu and Smith, 2013). In such contexts, therefore, some of the speech input that infants receive is naturally accompanied by visual input that is not cued by the speaker’s eye gaze. Hence, in this study, the ten-month-old infants might have attended equally to the speaker in the two conditions, because both events are perceived as equally likely to convey important linguistic input. Future studies might compare younger infants, who have less experience with triadic interactions, to older infants to observe whether the attention-holding effects of gaze change over development.

The lack of the attention-holding effects of gaze and the lack of a facilitatory effect of gaze on neural tracking might also partly be due to the screen-based design utilised in this study. We opted for a screen-based paradigm to standardise the acoustic signal over participants and conditions, as our measure of interest lied in the fine-grained temporal regularities of speech. The eye contact effect might have not come across as powerful with the screen-based design as eye contact in a live setting would, as infants may have paid more attention to the eye gaze cues of someone interacting with them live. Furthermore, real-life interactions contain an abundance of ostensive signals that accompany speaker’s gaze in different modalities, such as visual, tactile, and verbal cues. Therefore, having a live interlocutor interacting with the infant, as opposed to a video-recorded speaker, might have resulted in a larger effect, but it would be difficult to isolate an effect of eye gaze alone in such a setting.

It is also interesting to note that our coherence values were lower compared to studies that tested infants’ speech-brain coherence using live social interaction (Menn, Michel et al., 2022), while having yielded similar results to that of screen-based or auditory only paradigms with children (e.g. Ríos-López et al., 2020, Ríos-López et al., 2022) and adults (Vanden Bosch der Nederlanden et al., 2022). In fact, infants’ speech-brain coupling was overall higher in a live setting compared to the same stimuli presented over video, suggesting that live interaction might facilitate neural tracking of speech as well (Leong, Byrne et al., 2017), possibly by enhanced attention and arousal in live social interactions. Future work could compare live and screen-based presentations of ostensive naturalistic speech and its effect on infants’ neural tracking, with carefully controlled designs. Importantly, the multimodal and naturalistic nature of real-life caregiver-child interactions should be considered in order to have a clearer understanding of infants’ speech processing.

Finally, we found that infants’ neural tracking of audiovisual speech was a significant predictor of later language outcomes. In particular, we identified a link between subsequent expressive vocabulary and tracking at the syllable rhythm. Infants with stronger tracking at 10 months, especially at the syllable rate, produced more words at 18 months, controlling for their 10-month expressive vocabulary. Our results further corroborate recent findings suggesting links between infants’ tracking at the delta band (∼0.5–4 Hz), corresponding to the stress and at least partially to the syllable rhythm in typical infant-directed speech (Attaheri et al., 2022a, Attaheri et al., 2022b), as well as a link between tracking the stress rhythm and later vocabulary development (Menn, Ward et al., 2022). Successful tracking of the stress and syllable rate is argued to be relevant for language development, possibly because sensitivity to the important amplitude modulations around the stress and syllable rates in infant-directed speech may provide an advantage in segmenting word units from continuous speech, which, in turn, may predict later vocabulary development (Junge et al., 2012, Kidd et al., 2018, Kooijman et al., 2013). Thus, our results support the account that neural tracking may be an important building block for vocabulary development, such that infants with an early sensitivity to the structural units in language have larger later vocabularies.

While infants tracked the speech rhythm at both the stressed syllable and syllable rates, we only identified a relationship between speech-brain coherence at the syllable (but not stressed syllable) rate and expressive (but not receptive) vocabulary. The fact that we did not observe a relationship with receptive vocabulary may simply be because the receptive vocabulary scale is a noisier measure; parents are not as good at accurately recalling the words their children understand as they are at recalling the words their children say (see Fenson et al., 2007). The fact that we did not observe a relationship with the stressed syllable rate (contrary to previous studies; Attaheri et al., 2022a, Attaheri et al., 2022b; Menn, Ward et al., 2022) is harder to explain. One possible explanation is the slower speech rhythm in our stimuli (syllable rate: 2–5–3.5 Hz in our stimuli, compared to 3–5 Hz in Menn, Ward et al. (2022) who used Dutch nursery rhymes, and a mean articulation rate (excluding pauses) of approximately 4.5 Hz reported for Dutch IDS in Han et al. (2021) who found that Dutch parents slow down when talking about unfamiliar items). Our slower speech rhythm meant that the syllabic units also fell under the delta band frequency (0.5–4 Hz), which is the rate at which infants’ neural tracking was a predictor of later vocabulary in previous studies (Attaheri et al., 2022a, Attaheri et al., 2022b). In addition, our stimuli were audiovisual, which might also further support syllable tracking in particular. As in audiovisual speech, the visual speech cues (i.e. the articulatory movements of the speaker’s lips), correspond closely to the speech acoustic envelope at the syllable rate (Chandrasekaran et al., 2010), this correspondence may allow infants to form better temporal predictions about the speech rhythm and the transitional cues, thereby enhancing neural tracking. Therefore, tracking the syllable rhythm might be a more reliable cue in naturalistic interactions, where speech is typically audiovisual and multimodal. However, it should be noted that these interpretations are speculative, and further research is needed to understand whether stress and syllable tracking separately contribute to language development.

Overall, this study demonstrated that ten-month-old infants track the rhythm of naturalistic infant-directed speech at the stress and syllable rates. Infants’ neural tracking was not influenced by whether speech was conveyed with or without direct gaze, suggesting that the speaker’s use of direct gaze did not bring about a processing benefit for our sample of ten-month-old infants. Our results suggest that, in the early years, neural tracking might reflect a bottom-up stimulus-driven process, and that the speaker’s gaze does not influence neural tracking, at least not under ideal listening conditions. Future research is required to determine whether social cues are beneficial for infants’ neural tracking under more challenging listening conditions, such as speech-in-noise. Furthermore, neural tracking at the syllable rate was a significant predictor of later expressive vocabulary size at 18 months, indicating that early neural tracking abilities provide an advantage for building a vocabulary. These findings have implications for the functional role of neural tracking of speech, as well as for understanding the role of multimodal cues in face-to-face interactions.

## Funding statement

This work was funded by the 10.13039/501100004189Max Planck Society.

## Declaration of Competing Interest

The authors declare that they have no known competing financial interests or personal relationships that could have appeared to influence the work reported in this paper.

## Data Availability

The dataset generated and analysed during the current study will be made available on https://archive.mpi.nl/mpi/ for academic researchers upon publication of all articles based on the current study.
