# Supplementary material for: Ten-month-old infants’ neural tracking of naturalistic speech is not facilitated by the speaker’s eye gaze
Source: Dev Cogn Neurosci. 2023 Sep 29;64:101297. doi: 10.1016/j.dcn.2023.101297 (PMC10543766; doi:10.1016/j.dcn.2023.101297)
Supplement: Supplementary file 1 — Supplementary material [file mmc1.docx]

Supplementary Materials

1. Pre-registered analyses and deviations from the pre-registration

This study was preregistered (<https://aspredicted.org/blind.php?x=VSG_ZNB>). The preregistration also included an assessment of infants’ word segmentation abilities, but this is reported in a different paper (Çetinçelik et al., in submission). Here, we list any deviations from the pre-registered analyses and decisions pertaining to the current paper.

1. Exclusion criteria: In the preregistration, the criterion for exclusion based on the number of trials is listed as 20% of the total trials for the speech-brain coherence analyses, which amounts to approximately 180 trials. However, based on a tutorial paper by Bastos & Schoffelen (2016) which suggests that the sample size bias gets notably smaller around 50 trials, and based on current studies on infants’ speech-brain coherence which used much lower thresholds (e.g. 20 in Menn, Michel, et al., 2022; and 30 in Menn, Ward, et al., 2022), as well as a reviewer suggesting us to reconsider our strict threshold, we decided to lower our exclusion threshold to 30 per condition, meaning that we included infants who had 30 or more trials per condition (thus 60 trials overall).
2. Speech-brain coherence analyses: As there were no clear clusters of frequencies/electrodes showing overall speech-brain coherence (i.e. all electrodes and frequencies were significant between 1-10 Hz), we did not run linear-mixed effects models to compare the SBC for the direct and averted condition for specific clusters of frequencies/electrodes, but we used cluster-based permutation to assess condition differences in all electrodes and frequencies in one analysis (which was already indicated as an alternative in the pre-registration). Instead of 1-7 Hz, we analysed a slightly larger frequency range of 1-10 Hz.
3. Theta band power: In the pre-registration, we indicated that we will have baseline periods where the speaker’s face will be on the screen for about 2 seconds without any speech at the start of each block to control for visual effects of gaze. We did not have this baseline period at the start of each block (as that would increase the duration of the trials and reduce infants’ attentiveness), but only as two videos at the start of the experiment. We did not analyse this baseline period separately because not all infants had good data.
4. Note that the pre-registration indicates that the later vocabulary measures will be collected at 15 months, but this was corrected in an amended version of the pre-registration (<https://aspredicted.org/blind.php?x=ZY5_TFY>).
5. Looking Times in All Trials

A paired-samples t-test on infants’ mean looking times across all trials (including the trials contaminated with EEG artifacts and trials rejected based on looking, which were not included in the analyses) in the Direct and Averted gaze conditions showed no significant difference between the two conditions (*t*(49) = -0.95, *p* = .35; Direct: *M*= 2.26 s, *SD* = 1.67 s; Averted: *M*= 2.21 s, *SD* = 1.67 s).


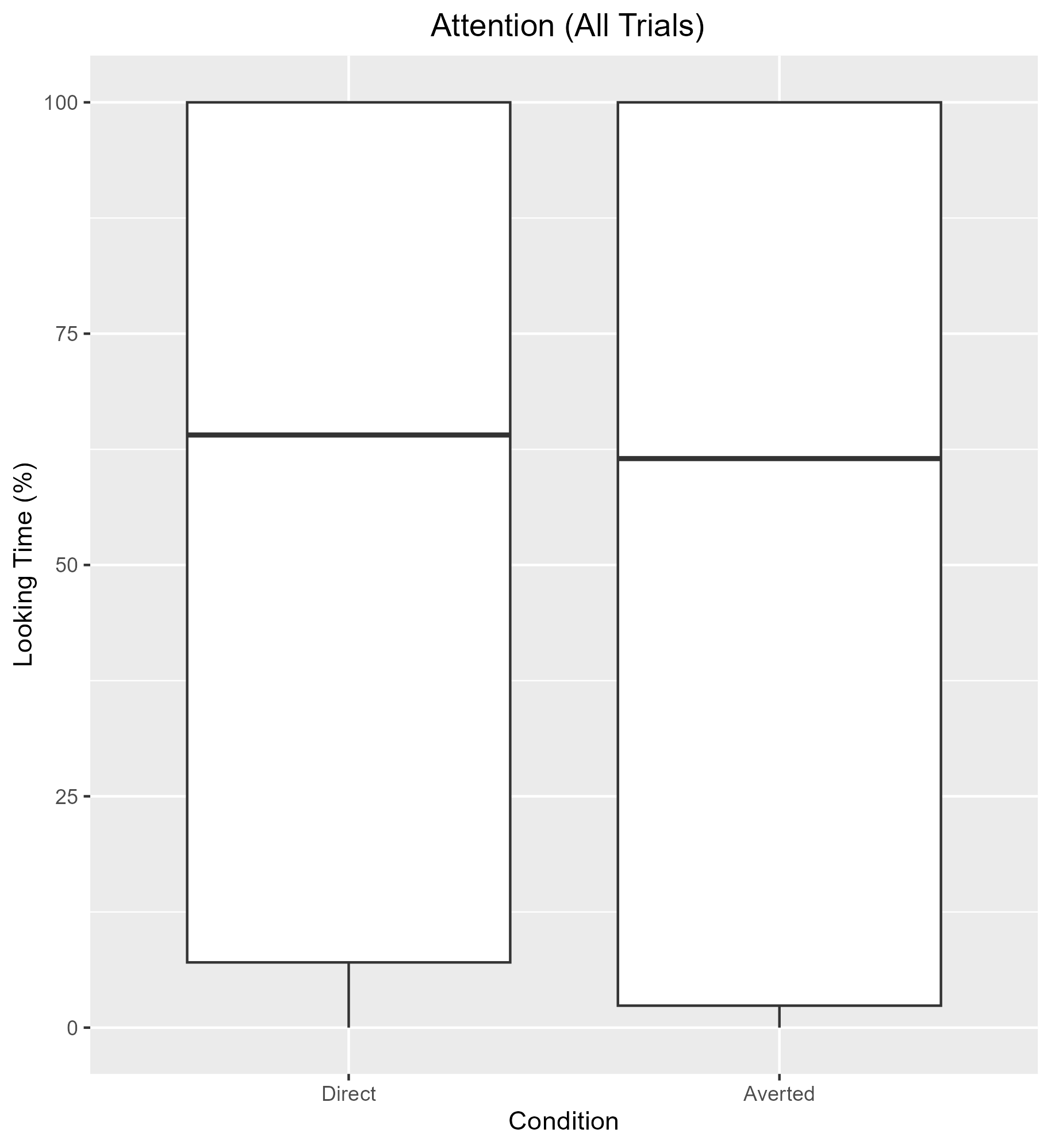


*Supplementary Figure 1.* Proportion of infants’ mean looking times (percentages, calculated by the average proportion of looking to the screen during each 4-second epoch) across all trials (including the trials contaminated with EEG artifacts and trials rejected based on looking, which were not included in the analyses) for the Direct and Averted gaze conditions. The length of the boxes represents the interquartile range, the whiskers show lower and upper values within 1.5 interquartile range. Note that all trials, including those without any looks at the screen, are plotted here, hence the possible minimum is 0%.

1. **Exploratory analysis with 50% attention**

In order to check whether our threshold of 25% attention per trial that was based on previous studies (Tan et al., 2022) was justified, we repeated our analyses including only trials with 50% and more attention per trial. With this exclusion criterion, 45 infants were included in the analyses (mean age = 308.08 days, range = 291-326; 25 females). In accordance with our main analyses, we tested for the presence of speech-brain coherence at the stress and syllable rates by comparing real SBC to surrogate SBC, as well as testing the difference in speech-brain coherence between the two conditions using cluster-based permutation tests.

Confirming our previous results, large positive clusters that encompassed all electrodes were identified at both the stress and syllable rates, both overall (*p_corrected_ =* .002; Supplementary Figure 2A) and in the two conditions (*p_corrected_ =* .002; Supplementary Figure 2B, left and middle columns). Testing for differences between the Direct and Averted gaze conditions, cluster-based permutation tests revealed no clusters (Supplementary Figure 2B, right column).

There were no significant differences in infants’ mean looking times between the two conditions (*t*(43) = 0.41, *p* = .88; Direct: *M*= 3.78 s, *SD* = .15 s; Averted: 3.79 s, *SD* = .16 s). Furthermore, we tested for differences between the two conditions in infants’ mean theta power (3-6 Hz) using cluster-based permutation, without averaging over the frequency range. Two positive and two negative clusters were identified, but were not significant after correcting for multiple comparisons (*p* of the largest positive cluster = .09, *p* of the largest negative cluster = .45).


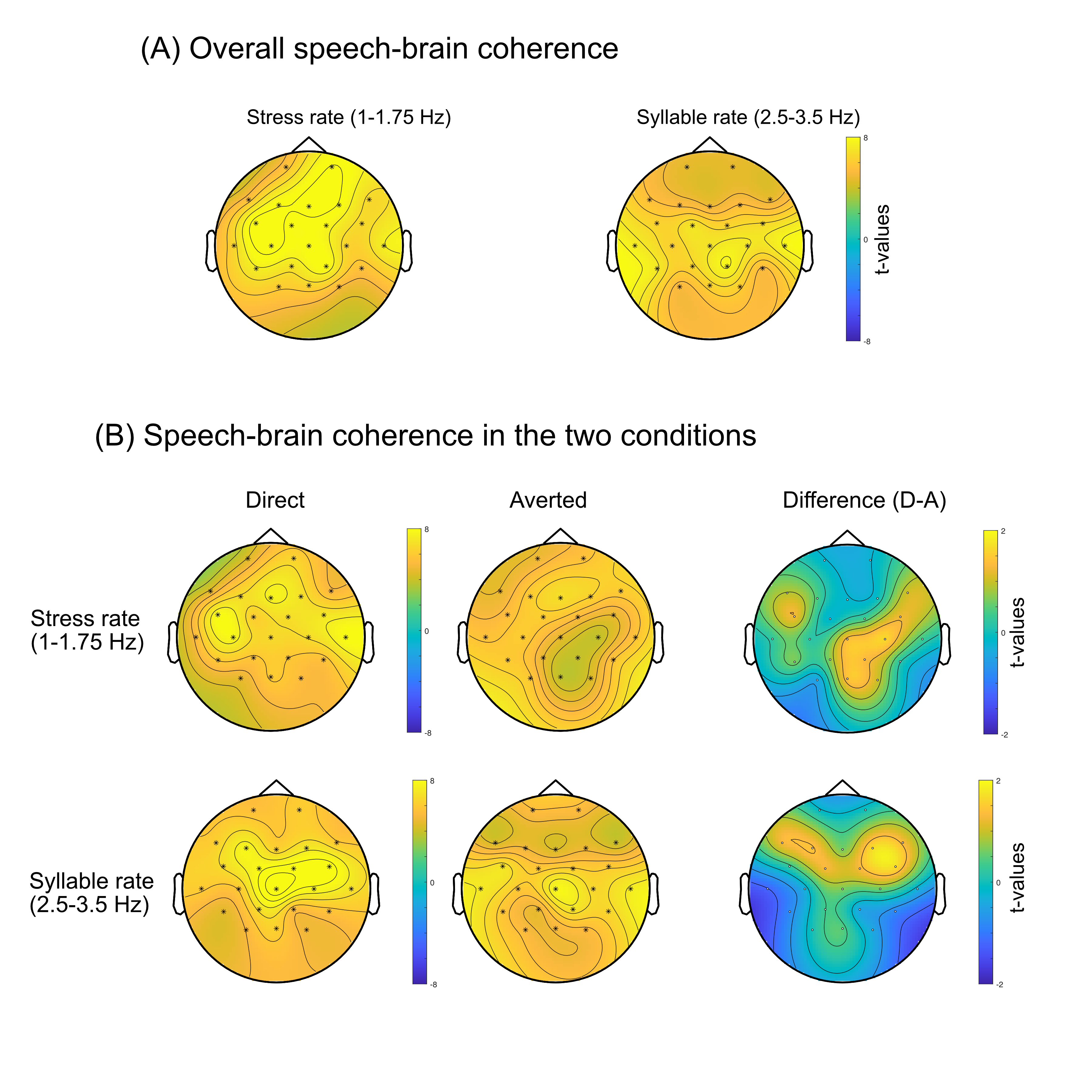


*Supplementary Figure 2.* Scalp topography speech-brain coherence, including only trials with 50% and higher attention. (A) Overall speech-brain coherence in the stress and syllable rates, showing the t-values of the comparison. (B) Speech-brain coherence in the Direct gaze (left column), Averted gaze conditions (middle column) and the difference between the Direct and Averted gaze conditions (right column). The top row illustrates coherence at the stress rate (1-1.75 Hz), and the bottom row illustrates coherence at the syllable rate (2.5-3.5 Hz). The topographies in the left and middle columns show the t-values of the comparison between real versus surrogate data in the two conditions, and the right column shows the comparison of the Direct and Averted conditions. Note the different scales used for the colour bar in the left and middle columns (t-values between -8 and 8) and the difference figure on the right (t-values between -2 and 2). Cluster electrodes involved in the significant clusters for real versus surrogate data (in 2A and left and middle columns in 2B) are marked with stars.

1. **Stimulus Materials**

*Supplementary Table 1.* The full set of stimulus materials.

| Block | Target words | Familiarisation sentences |
| --- | --- | --- |
| 1 | cello’s/tuba’s/banjo’s | Er zitten cello’s/tuba’s/banjo’s in het orkest. |
|  |  | Goede cello’s/tuba’s/banjo’s zijn van hout gemaakt. |
|  |  | Ik hoorde vanochtend cello’s/tuba’s/banjo’s. |
|  |  | Met de pauken spelen vaak de cello’s/tuba’s/banjo’s mee. |
| 2 | gamba/oester/scampi | In dure restaurants kun je gamba/oester/scampi eten. |
|  |  | Bij champagne eet je lekker een gamba/oester/scampi. |
|  |  | Na het zwemmen rust de gamba/oester/scampi uit. |
|  |  | Iedere gamba/oester/scampi leeft in zout water. |
| 3 | kwartel/eland/marter | Ik hoorde laatst een echte kwartel/eland/marter roepen. |
|  |  | Bij de boom is een grote kwartel/eland/marter. |
|  |  | Een koude sneeuwbal vindt kwartel/eland/marter geen probleem. |
|  |  | De lieve kwartel/eland/marter kan goed tegen kou. |
| 4 | bellers/piefen/zwetsers | Die leuke bellers/piefen/zwetsers praten als de beste. |
|  |  | Dat moet ik overleggen met de bellers/piefen/zwetsers. |
|  |  | De grootste bellers/piefen/zwetsers wonnen een prijs. |
|  |  | Na het werk gingen de laatste bellers/piefen/zwetsers naar huis. |
| 5 | havik/arend/buizerd | Duif en havik/arend/buizerd kijken wie er het beste vliegt. |
|  |  | In Limburg was een mooie havik/arend/buizerd. |
|  |  | Deze havik/arend/buizerd heeft zachte veren. |
|  |  | Muizen vangen vindt de havik/arend/buizerd leuk. |
| 6 | sesam/dadel/bieslook | Op een bagel is sesam/dadel/bieslook erg lekker. |
|  |  | Ik hou van lekkere sesam/dadel/bieslook. |
|  |  | In het zuiden wordt sesam/dadel/bieslook verbouwd. |
|  |  | Als je sesam/dadel/bieslook perst dan krijg je olie. |
| 7 | bloesem/klaver/rogge | Er lag een hoopje bloesem/klaver/rogge naast de eik. |
|  |  | In de lente is er overal bloesem/klaver/rogge. |
|  |  | Je kunt bloesem/klaver/rogge mooi op tafel leggen. |
|  |  | Er ligt wat bloesem/klaver/rogge in het gras. |
| 8 | buffel/veulen/tarpan | Wat heeft jouw buffel/veulen/tarpan een zachte vacht! |
|  |  | Na heel ver rennen was hun buffel/veulen/tarpan moe. |
|  |  | Maartjes buffel/veulen/tarpan staat altijd buiten. |
|  |  | Koe stond samen met buffel/veulen/tarpan in de wei. |
| 9 | doffers/gieren/spreeuwen | Door te vliegen hebben doffers/gieren/spreeuwen sterke vleugels. |
|  |  | Dat zijn hele lieve doffers/gieren/spreeuwen. |
|  |  | Deze doffers/gieren/spreeuwen lusten alles! |
|  |  | Samen reizen vinden doffers/gieren/spreeuwen het leukst. |
| 10 | toekans/spechten/reigers | De snavels van toekans/spechten/reigers zijn heel groot. |
|  |  | Kijk, daar zitten mooie toekans/spechten/reigers! |
|  |  | Samen in een nestje zijn toekans/spechten/reigers erg lief. |
|  |  | In het oerwoud kun je toekans/spechten/reigers zien. |
| 11 | dille/venkel/lavas | Ik doe altijd wat dille/venkel/lavas op mijn vis. |
|  |  | Duitse koks koken graag met dille/venkel/lavas. |
|  |  | Wij hebben dille/venkel/lavas in de tuin. |
|  |  | Zullen we in de salade wat dille/venkel/lavas doen? |
| 12 | kreeften/wulken/pieren | De zee vinden kreeften/wulken/pieren het leukst. |
|  |  | Wat een mooie kreeften/wulken/pieren zijn dat zeg! |
|  |  | Onder water vind je kleine kreeften/wulken/pieren |
|  |  | Restjes eten is voor kreeften/wulken/pieren heel lekker. |
| 13 | bode/pachter/reder | In de regen is de bode/pachter/reder niet zo blij. |
|  |  | Er past veel in de tas van die bode/pachter/reder. |
|  |  | Onze bode/pachter/reder is altijd op tijd. |
|  |  | De brieven van zijn bode/pachter/reder liggen in de la. |
| 14 | sloeber/doerak/looier | Die goeie sloeber/doerak/looier geeft vaak feestjes. |
|  |  | Er zat een vieze sloeber/doerak/looier bij het vuur. |
|  |  | Dat was stout van die stomme sloeber/doerak/looier. |
|  |  | De lieve sloeber/doerak/looier deed wel zijn best. |
| 15 | vorsten/heersers/sultans | Vroeger waren vorsten/heersers/sultans vaak heel jong. |
|  |  | Mensen met geld leven als vorsten/heersers/sultans. |
|  |  | Alle vorsten/heersers/sultans zitten op een troon. |
|  |  | De dochters van vorsten/heersers/sultans zijn prinsessen. |
| 16 | karper/paling/adder | Onze kleine karper/paling/adder leeft in de sloot. |
|  |  | Ik zag laatst een hele grote karper/paling/adder! |
|  |  | Kan deze karper/paling/adder ook in zout water zwemmen? |
|  |  | Door vervuiling wordt de karper/paling/adder bedreigd. |
| 17 | kwallen/algen/riffen | Zijn er ook kwallen/algen/riffen onderwater? |
|  |  | Sommige kwallen/algen/riffen geven mooi licht. |
|  |  | Bij dat meer zijn allemaal mooie kwallen/algen/riffen! |
|  |  | Een paar gemene kwallen/algen/riffen zijn heel gevaarlijk. |
| 18 | bami/selder/foelie | Daar zou ik wat bami/selder/foelie bij doen. |
|  |  | In China eten ze heel vaak bami/selder/foelie. |
|  |  | Je kan ook bami/selder/foelie bij de soep eten. |
|  |  | Zou Surinaamse bami/selder/foelie anders smaken? |
| 19 | hommels/kevers/torren | Die dikke hommels/kevers/torren zitten graag op bloemen. |
|  |  | Mijn favoriete insecten zijn hommels/kevers/torren. |
|  |  | De mooiste hommels/kevers/torren vliegen buiten. |
|  |  | Hebben hommels/kevers/torren ook een koningin? |
| 20 | krokus/anjer/dotter | Die kleine krokus/anjer/dotter staat in de zon. |
|  |  | Vandaag plant ik denk ik een krokus/anjer/dotter. |
|  |  | Wat een mooie krokus/anjer/dotter is dat! |
|  |  | Die lieve krokus/anjer/dotter staat naast een narcis. |
| 21 | egel/made/poema | Onze egel/made/poema leeft in de tuin. |
|  |  | Op die boomstronk zit een grote egel/made/poema! |
|  |  | Wij hielpen die lieve egel/made/poema wel. |
|  |  | Kattenvoer lust een egel/made/poema ook! |
| 22 | hinde/bever/zwaluw | Die mooie hinde/bever/zwaluw is bij de vijver. |
|  |  | In het bos zag ik laatst een hinde/bever/zwaluw. |
|  |  | Het lievelingseten van hinde/bever/zwaluw is boomschors. |
|  |  | Naast de kleine hinde/bever/zwaluw lag een vos. |
| 23 | gondels/schuiten/prauwen | Zulke gondels/schuiten/prauwen bestuur je met een stok. |
|  |  | In Venetië zie je veel kleine gondels/schuiten/prauwen. |
|  |  | In kanalen kun je soms gondels/schuiten/prauwen zien. |
|  |  | Met die gondels/schuiten/prauwen kun je het water over gaan. |
| 24 | cantor/blazer/oempa | In het orkest speelt die cantor/blazer/oempa altijd mee. |
|  |  | De zangers kunnen niet spelen zonder cantor/blazer/oempa. |
|  |  | De band lachte altijd de cantor/blazer/oempa uit. |
|  |  | Zou hun cantor/blazer/oempa goed kunnen zingen? |
| 25 | gieters/silo’s/mossen | Die winkel biedt allerlei gieters/silo’s/mossen aan. |
|  |  | Boeren houden van grote gieters/silo’s/mossen. |
|  |  | Langs de weg zag Pietje gieters/silo’s/mossen staan. |
|  |  | Die grote gieters/silo’s/mossen vallen wel op zeg! |
| 26 | krekels/hoenders/drongo’s | Het lijkt net of die krekels/hoenders/drongo’s een liedje zingen. |
|  |  | Julia houdt niet van gemene krekels/hoenders/drongo’s. |
|  |  | Daar zitten mooie krekels/hoenders/drongo’s in het gras. |
|  |  | De kippen doen de krekels/hoenders/drongo’s na. |
| 27 | mammoet/gramper/orka | Die grote mammoet/gramper/orka houdt wel van een ijsje. |
|  |  | Wat een boel littekens heeft die mammoet/gramper/orka! |
|  |  | Heb jij wel eens een mammoet/gramper/orka gezien? |
|  |  | Die mooie mammoet/gramper/orka kan goed fluiten. |
| 28 | frater/prior/goeroe | De gelovige frater/prior/goeroe zit op een steen. |
|  |  | Die Bijbel is van die lieve frater/prior/goeroe. |
|  |  | Draagt een frater/prior/goeroe ook een hoedje? |
|  |  | Die kleine frater/prior/goeroe hoort bij een klooster. |
| 29 | garum/soja/ketjap | Kaas met garum/soja/ketjap is erg lekker. |
|  |  | Het lekkerst is zelfgemaakte garum/soja/ketjap. |
|  |  | Is die garum/soja/ketjap voor bij de sushi? |
|  |  | Voor mij is garum/soja/ketjap veel te sterk. |
| 30 | kollen/feeksen/gnomen | Blijkbaar dragen kollen/feeksen/gnomen vaak hoedjes! |
|  |  | De groep kollen/feeksen/gnomen en de reus liepen naar de gracht. |
|  |  | In het sprookjesbos wonen kollen/feeksen/gnomen. |
|  |  | Langzaam sjokten de kollen/feeksen/gnomen naar de prins. |

Supplementary Materials References

Bastos, A. M., & Schoffelen, J.-M. (2016). A Tutorial Review of Functional Connectivity Analysis Methods and Their Interpretational Pitfalls. *Frontiers in Systems Neuroscience*, *9*. https://www.frontiersin.org/articles/10.3389/fnsys.2015.00175

Menn, K. H., Michel, C., Meyer, L., Hoehl, S., & Männel, C. (2022). Natural infant-directed speech facilitates neural tracking of prosody. *NeuroImage*, *251*, 118991. https://doi.org/10.1016/j.neuroimage.2022.118991

Menn, K. H., Ward, E. K., Braukmann, R., van den Boomen, C., Buitelaar, J., Hunnius, S., & Snijders, T. M. (2022). Neural Tracking in Infancy Predicts Language Development in Children With and Without Family History of Autism. *Neurobiology of Language*, *3*(3), 495–514. https://doi.org/10.1162/nol_a_00074

Tan, S. H. J., Kalashnikova, M., Di Liberto, G. M., Crosse, M. J., & Burnham, D. (2022). Seeing a talking face matters: The relationship between cortical tracking of continuous auditory‐visual speech and gaze behaviour in infants, children and adults. *NeuroImage*, *256*, 119217. https://doi.org/10.1016/j.neuroimage.2022.119217
